# New Insight Into the Interspecies Shift of Anammox Bacteria *Ca.* “Brocadia” and *Ca.* “Jettenia” in Reactors Fed With Formate and Folate

**DOI:** 10.3389/fmicb.2021.802201

**Published:** 2022-02-03

**Authors:** Anna Kallistova, Yury Nikolaev, Vladimir Grachev, Alexey Beletsky, Evgeny Gruzdev, Vitaly Kadnikov, Alexander Dorofeev, Julia Berestovskaya, Anna Pelevina, Ivar Zekker, Nikolai Ravin, Nikolai Pimenov, Andrey Mardanov

**Affiliations:** ^1^Research Center of Biotechnology, Russian Academy of Sciences, Moscow, Russia; ^2^Institute of Chemistry, University of Tartu, Tartu, Estonia

**Keywords:** anammox bacteria, *Candidatus* “Brocadia”, *Candidatus* “Jettenia”, formate, folate, interspecies shift

## Abstract

The sensitivity of anaerobic ammonium-oxidizing (anammox) bacteria to environmental fluctuations is a frequent cause of reactor malfunctions. It was hypothesized that the addition of formate and folate would have a stimulating effect on anammox bacteria, which in turn would lead to the stability of the anammox process under conditions of a sharp increase in ammonium load, i.e., it helps overcome a stress factor. The effect of formate and folate was investigated using a setup consisting of three parallel sequencing batch reactors equipped with a carrier. Two runs of the reactors were performed. The composition of the microbial community was studied by the 16S rRNA gene profiling and metagenomic analysis. Among anammox bacteria, *Ca.* “Brocadia” spp. dominated during the first run. A stimulatory effect of folate on the daily nitrogen removal rate (dN) was identified. The addition of formate led to progress in dissimilatory nitrate reduction and stimulated the growth of *Ca.* “Jettenia” spp. The spatial separation of two anammox species was observed in the formate reactor: *Ca.* “Brocadia” occupied the carrier and *Ca.* “Jettenia”—the walls of the reactors. Biomass storage at low temperature without feeding led to an interspecies shift in anammox bacteria in favor of *Ca.* “Jettenia.” During the second run, a domination of *Ca.* “Jettenia” spp. was recorded along with a stimulating effect of formate, and there was no effect of folate on dN. A comparative genome analysis revealed the patterns suggesting different strategies used by *Ca.* “Brocadia” and *Ca.* “Jettenia” spp. to cope with environmental changes.

## Introduction

Anaerobic ammonium-oxidizing (anammox) bacteria carry out anoxic oxidation of ammonium with nitrite to dinitrogen gas coupled with the production of small amounts of nitrate (Strous et al., 1998). These bacteria play an important role in the global nitrogen cycle and are used in biotechnology for ammonium removal from wastewater (Kartal et al., 2004; Kuenen, 2020). The sensitivity of anammox bacteria to changes in temperature; pH; and concentrations of dissolved oxygen (DO), ammonium, nitrites, sulfides, and organic compounds is a frequent cause of reactor failures in actual conditions of wastewater treatment facilities (Lackner et al., 2014; Cho et al., 2020). In this respect, it is essential to look for metabolic effectors (regulators) that would allow the anammox community to cope with unfavorable fluctuations in external conditions and help maintain the stability of nitrogen removal from wastewater.

Volatile fatty acids (VFAs) such as formate, acetate, and propionate could serve as potential regulators of the activity of the anammox community. Anammox bacteria can directly assimilate VFAs (Feng et al., 2019; Zhang et al., 2021) and improve their nitrogen metabolism by coupling anaerobic VFA oxidation with the process of dissimilatory nitrate reduction (DNRA). The nitrate reduction by anammox bacteria was shown to be coupled with the oxidation of formate, where nitrate was the electron acceptor and formate—the electron donor. In the DNRA process, anammox bacteria form nitrite and ammonium from nitrate and then use these compounds in a classic chemolithoautotrophic anammox process. VFA oxidation coupled with nitrate reduction also helps anammox bacteria survive under conditions of ammonium and nitrite limitation and reduces their dependence on microorganisms that form these compounds (Kartal et al., 2007). While anammox bacteria grow well with nitrate as the sole nitrogen source, their ability to perform a complete DNRA process was questioned in the present study. Anammox bacteria were shown to express the *narG* gene for the reduction of nitrate to nitrite in Fe^0^/Fe^2+^-dependent partial DNRA, whereas ammonium was produced from nitrite by abiotic reduction, facilitated by Fe^0^ and Fe^2+^ (Bi et al., 2020).

When determining the rate of VFA oxidation by various anammox bacteria (*Ca.* “Brocadia,” *Ca.* “Kuenenia,” and *Ca.* “Anammoxoglobus”), the rate of formate oxidation was an order of magnitude higher than that of acetate and propionate in all studied cultures and was maximal for *Ca.* “Brocadia fulgida” (Kartal et al., 2007, 2008). It was demonstrated that the anammox bacterium *Ca.* “K. stuttgartiensis” was able to assimilate formate directly *via* the reductive acetyl-CoA pathway. However, it was not able to use acetate as a carbon or energy source, and the oxidation of acetate was carried out by other bacteria of the bioreactor’s community (Lawson et al., 2020). In contrast to the above-mentioned anammox bacteria, the activity of *Ca.* “Jettenia” was shown to be inhibited by formate in a concentration of 1 mM (corresponding to 68 mg/l in a form of sodium salt), but not by acetate (Ali et al., 2015). No significant difference in anammox activity was observed with an addition of formate in the concentration range of 25–100 mg/l; it slightly exceeded the activity in the control. Regarding acetate supply, an increase in its concentration to 50 mg/l led to a slight increase in anammox activity, while at a further increase (from 50 to 100 mg/l), the anammox activity was inhibited (Wang L. et al., 2021). The VFA addition at certain concentrations could be beneficial for biotechnology as it promotes higher removal of nitrogen (including nitrate) and organic contaminants and the stabilization of the process (Kartal et al., 2007; Feng et al., 2019; Yin et al., 2019; Li et al., 2020; Zhang et al., 2021). The inhibitory effect of formate on undesirable nitrite-oxidizing bacteria was additionally found (Wang et al., 2020).

Secondary metabolites could be other potential regulators of the activity of the anammox community. Secondary metabolites are not directly required for microbial growth but are important for many biological processes, especially those involved in microbial interactions. The CO_2_ fixation in anammox bacteria involves a folate-dependent, one-carbon metabolic pathway (the Wood–Ljungdahl route) (Jetten et al., 2009; Kartal et al., 2013). Folate is a necessary subunit for anammox CO_2_ fixation, including 10-formyltetrahydrofolate, 5,10-methenyltetrahydrofolate, and 5,10-methylenetetrahydrofolate, and it is therefore vital for anammox growth and activity (Wang et al., 2016; Zhao et al., 2018). At the same time, anammox bacteria are not capable of performing their own biosynthesis. Therefore, they have to borrow it from folate-producing prokaryotes as it is the secondary metabolite of many bacterial species. It has been shown that the growth and activity of folate-deficient bacteria increase after co-cultivation with folate-producing bacteria (Leduc et al., 2004). Folate cross-feeding between bacteria of the phyla *Armatimonadetes*, *Proteobacteria*, and *Planctomycetes* (anammox bacteria) was described. It was assumed that such cross-feedings could improve the nitrogen removal rate by increasing anammox growth and activity (Zhao et al., 2018).

The aim of this work was to study the effect of formate and folate on the activity and composition of the anammox community under conditions of sharply increased ammonium loads. Formate serves as an additional substrate for anammox bacteria. Folate being a secondary metabolite of *Proteobacteria*, can potentially increase the rate of nitrogen removal. Thus, it was hypothesized that the addition of these compounds would have a stimulatory effect on the anammox community enriched with *Ca.* “Brocadia,” which in turn would lead to the stability of the anammox process under conditions of increased ammonium load, i.e., it would help overcome a stress factor. After the experiment was completed, a mixed biomass sample was stored at +4°C for 3 months, and an interspecies shift between anammox bacteria from *Ca.* “Brocadia” to *Ca.* “Jettenia” was further discovered. Interspecies shifts between different anammox bacteria have been frequently observed in laboratory reactors [reviewed by Zhang and Okabe (2020)]. This suggested the existence of genus- or species-specific niche differentiation and competition between anammox bacteria caused by differences in maximum specific growth rates, affinities to limiting substrates (ammonium and nitrite), susceptibility to various compounds, or VFA utilization (Zhang et al., 2021). However, the data explaining the niche differentiation of anammox bacteria are often contradictory, and reasons for one anammox genera or species outcompeting another are still largely unknown (Ali et al., 2018; Zhang and Okabe, 2020). In the present study, the second run of the reactors was performed to find out if the effect of formate and folate on the new *Ca.* “Jettenia”-enriched anammox community is different from the *Ca.* “Brocadia”-enriched one and to reveal the probable causes of an interspecies shift by applying a comparative genome analysis.

## Materials and Methods

### Setup Configuration and Operation

**FIGURE 1:**
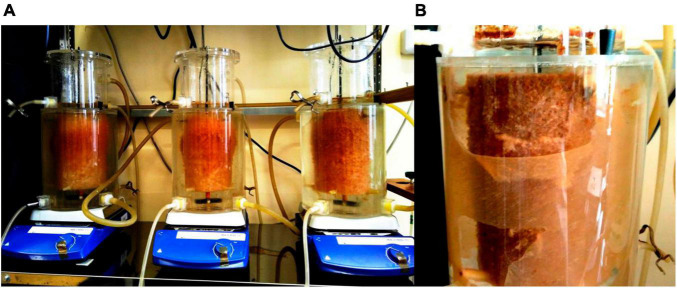
A setup configuration 3 weeks after a start-up **(A)** and two types of the activated sludge biofilms: on the carrier (carrier) and the inner part of the walls of the reactor (glass) in the formate reactor during the first run (107th day) **(B)**.

A setup consisted of three sequencing batch reactors (SBRs) operated in parallel, working with complete biomass retention on a fibrous porous carrier (Figure 1A). A detailed design of each reactor was described in Kallistova et al. (2020). Each reactor performed a single-stage partial nitritation/anammox process. The operating mode included a 20-min settling stage, a 30-min stage of feeding by a synthetic medium with simultaneous effluent drainage, and a 310-min stage of aeration and mechanical stirring alternated every 20 min with a 10-min duration of the last stirring phase. The complete cycle duration was 6 h, and the hydraulic retention time (HRT) was 27 h. The operating temperature was 32 ± 2°C, pH was 8.3 ± 0.1, and DO was 0.6 ± 0.2 mg/l. The airflow was gradually increased from 20 ± 0.5 to 30 ± 0.5 l/h during the entire period of the setup operation. The medium composition and preparation are described in Kallistova et al. (2020). An activated sludge containing anammox bacteria (Mardanov et al., 2019a) was used as an inoculum for the reactors’ first run. One liter of the *inoculum* with a total suspended solid content of 3.8 g/l was added to 3.5 L of the medium to start each reactor. Nitrite was not added to the medium as it was produced in the process of stage I nitrification. The ammonium concentration was increased from 200 to 400 mg N–NH_4_/l in all reactors after the nitrogen removal efficiency reached >80% (65th day). Formate (in the form of sodium salt) was added to the first reactor at a concentration of 75.5 mg/l, and folate (0.2 mg/l) was added to the second parallel reactor; the third reactor remained the control with no additives.

The reactors were stopped after 1.5 months after the additives were supplied (on the 110th day). A sample of biomass mixed from three reactors was stored at +4°C for 3 months. The storage conditions were chosen taking into account the recommendations of Ji and Jin (2014). This biomass was used after the storage to re-inoculate reactors with fresh media (the second run). Formate and folate were added immediately after the inoculation. The ammonium concentration was increased in all reactors from 200 to 500 mg N–NH_4_/l on the 106th day.

### Analytical Methods

Concentrations of ammonium, nitrite, and nitrate were determined in the effluent using the standard methods (Rice and Bridgewater, 2012). The daily amount of removed nitrogen (dN, milligrams per liter) was calculated as the difference between the concentration of ammonium nitrogen (N–NH_4_) in the inflowing medium and the concentrations of mineral nitrogen species (N–NH_4_, N–NO_2_, and N–NO_3_) in the effluent. The efficiency of nitrogen removal (%) was calculated as the share of removed nitrogen of its concentration in the influent.

### Statistical Analysis

Mean values, standard deviations, and Student’s *t*-test were calculated in MS Excel. The experimental error of nitrogen mineral form concentrations (calculated as the mean deviations of experimental values from the mean value) did not exceed 2.7%.

### 16S rRNA Sequencing and Analysis

High-throughput sequencing of 16S rRNA gene fragments was used to analyze the community composition in two types of activated sludge biofilms: in biofilms attached to the carrier (further referred to as carrier) and at the inner part of the walls of the reactors (further referred to as glass) in the experimental (in the presence of formate and folate) and in the control reactors (Figure 1B).

Metagenomic DNA was isolated from biofilm samples using the DNeasy Power-Soil Kit (Qiagen, Germany) according to the manufacturer’s recommendations. Two sets of primers were used to amplify the V3–V4 variable region of the 16S rRNA gene: universal primers 341F (CCTAYGGGDBGCWSCAG) and 806R (GGACTACNVGGGTHTCTAAT) (Frey et al., 2016) and specific primers for anammox bacteria, Amx368f (TTCGCAATGCCCGAAAGG) and Amx820r (GGGCACTAA GTAGAGGGGTTTT) (Sonthiphand and Neufeld, 2013). The obtained PCR fragments were used to prepare the libraries with the Nextera XT DNA Library Prep Kit (Illumina, United States) according to the manufacturer’s recommendations. Multiplexing was carried out using the Nextera XT Index Kit v2 (Illumina, United States). PCR fragments were sequenced using Illumina MiSeq. At least 25,000 sequences of the 16S rRNA gene fragments were obtained for each sample. Reads from all samples were pooled together; low-quality reads, singletons, and chimeras were excluded from the analysis. The remaining reads were clustered into operational taxonomic units (OTUs) with a minimal identity of 97%. To determine the shares of OTUs in each sample, original reads (including low-quality and singleton ones) with a minimal identity of 97% were superimposed over the representative OTU sequences. These procedures were carried out using the USEARCH software package (Edgar, 2010). Taxonomic identification based on the 16S rRNA gene sequences was carried out using USEARCH and the SILVA database.

### Metagenome Sequencing and Analysis

A combination of Illumina and Oxford Nanopore high-throughput sequencing technologies was used for sequencing the metagenome. At the first stage, the sequencing of a paired-end (2 × 150 bp) NEBNext^®^ Ultra™ II DNA Library using an Illumina HiSeq2500 platform (Illumina, San Diego, CA, United States) resulted in the acquisition of 69,080,752 read pairs. In the obtained reads, the adapter removal and trimming of low-quality sequences (*Q* < 30) were performed using Cutadapt v.1.8.3 (Martin, 2011) and Sickle v.1.33 (Joshi and Fass, 2011), respectively. Trimmed reads were merged using FLASH v.1.2.11 (Magoč and Salzberg, 2011). The resulting merged and unmerged reads (about 20.7 Gbp in total) were *de novo* assembled into contigs using metaSPAdes genome assembler v.3.13.0 (Bankevich et al., 2012).

Contigs longer than 1,500 bp were binned into clusters representing metagenome-assembled genome (MAGs) using CONCOCT v.0.4.1 (Alneberg et al., 2014) and MetaBAT v.2.12.1 (Kang et al., 2015). The completeness of the MAGs and their possible contamination (redundancy) were estimated using CheckM v.1.05 (Parks et al., 2015) with lineage-specific marker genes. The assembled MAGs were taxonomically classified using the Genome Taxonomy Database Toolkit (GTDB-Tk) v.0.3.2 and Genome Taxonomy Database (GTDB) (Parks et al., 2018). The contig-to-MAG binning scheme was selected manually by comparing the results from MetaBAT and CONCOCT.

For Nanopore sequencing, the library was prepared using the 1D ligation sequencing kit (SQK-LSK109, Oxford Nanopore, United Kingdom). The sequencing of this library in an R10 flow cell (FLO-MIN110) yielded 788,000 reads with a total length of 3.1 Gbp using the MinION device. Hybrid assembly of Illumina and Nanopore reads was performed using Unicycler v. 0.4.8 (Wick et al., 2017).

Gene search and annotation of MAGs were performed using RAST server 2.0 (Brettin et al., 2015), followed by manual correction of the annotation by comparing the predicted protein sequences with the NCBI databases.

### Network Analysis

Co-occurrence networks were inferred based on a Spearman correlation matrix (Langfelder and Horvath, 2012) and constructed using only significant correlation (Barberán et al., 2014). Only OTUs, the relative abundance of which was at least 2% in at least one sample, were included in the analysis. The cutoff for correlation coefficients was determined to be 0.6, and the cutoff for adjusted *p*-values was 0.001 (Luo and Bhattacharya, 2006). Visualization of co-occurrence network was performed using the Cytoscape v.3.8.2 platform (Shannon et al., 2003; Faust and Raes, 2016).

## Results

### The First Run of the Reactors

#### Dynamics of the Concentration of Mineral Nitrogen Species

**FIGURE 2:**
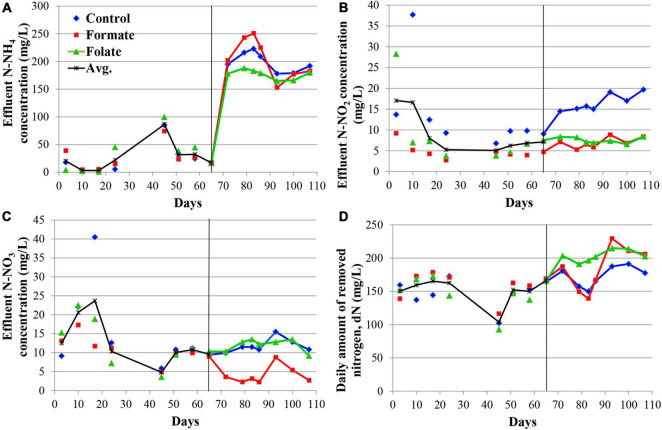
Dynamics of the concentrations of nitrogen compounds in the effluent during the first run of the reactors (milligrams per liter): concentrations of N–NH_4_ **(A)**, N–NO_2_ **(B)**, N–NO_3_ **(C)**, and daily amount of removed nitrogen (dN) **(D)**. A black vertical line separates periods before and after the additives supplied and the ammonium load increased. The data for each reactor are presented as colored dots and lines; a black curve (avg., average) reflects the values averaged for all three reactors.

Figure 2 presents the dynamics of the concentration of mineral nitrogen compounds in the effluent. For the first 2 months, the reactors were operated in the same mode (without the supply of the additives), which can be considered as three independent replicates of one experiment. The concentration of the incoming ammonium was maintained during this period at a constant level of 200 mg N–NH_4_/l. The ammonium concentration in the effluent varied in the range of 3–86 mg N–NH_4_/l (Figure 2A). Such fluctuations are associated with the commissioning stage, i.e., the attachment of the biomass on the carrier and adaptation of the community to new conditions. Nitrite was not added to the medium; however, it was present in the effluent, which indicated the occurrence of stage I nitrification in the reactors. For the three reactors observed, the average concentration of nitrite in the effluent varied during the first 2 months of operation in the range of 5–17 mg N–NO_2_/l (Figure 2B) and that of nitrate in the range of 5–24 mg N–NO_3_/l (Figure 2C). Elevations in the concentrations of nitrite (37.7 mg N–NO_2_/l) on the 10th day and nitrate (40.5 mg N–NO_3_/l) on the 17th day were recorded in the reactor, which later became the control one. These were the maximum values of nitrite and nitrate concentrations for the entire experiment and may indicate an imbalance between stages I and II nitrifiers and anammox bacteria in the community of this reactor. Since the dN did not decrease in this period, and the amount of ammonium in the effluent did not increase, it can be assumed that total nitrogen removal occurred in the future control reactor due to the process of nitri-denitrification, rather than nitritation-anammox. The dN in the reactors varied in the range of 103–166 mg N/l (mean 151.2 ± 20.25 mg N/l) (Figure 2D). The reactors removed 166.3 mg N/l by the 65th day, which corresponded to an ammonium removal efficiency of 83%.

The nitrogen load was increased from 177.8 to 355.6 mg/l/day in all reactors after the 65th day by increasing the ammonium concentration from 200 to 400 mg N–NH_4_/l. At the same time, formate was added to the first reactor and folate to the second one; no additives were supplied to the control reactor. The average dN, starting from the 65th day, were 172.7 ± 15.6 mg N/l (in the control), 184.1 ± 33.5 mg N/l (in the formate reactor), and 203.4 ± 8.6 mg N/l (in the folate reactor), which corresponded, however, to a lower overall process efficiency compared to the period before the load increased (40–43 vs 83%). The addition of formate led to a decrease in the nitrate concentration in the effluent from 11 to 4 mg N–NO_3_/l, which was three times lower than that in the control and in the folate reactors and could be associated with the process of nitrate reduction with formate (DNRA) by anammox bacteria. The nitrite content increased significantly in the control reactor from 9.5 to 19.7 mg N–NO_2_/l, while in both experimental reactors, this indicator did not change compared to the period before the supply of the additives (staying 7.0 ± 1.3 and 7.6 ± 0.7 mg N–NO_2_/l in the formate and folate reactor, respectively). In the control reactor, the impact of the stage I nitrifiers and denitrifiers probably increased, and the role of anammox bacteria decreased due to failures at the start-up stage.

In general, the dN was higher for the reactor with the addition of folate than with formate. Folate stimulated the process of nitrogen removal by 18% compared to the control. This stimulation started from day one after its addition, while the effect of formate (dN was 7–11% higher in the formate reactor compared to the control one) manifested itself only after 3 weeks. It has to be noted that a difference of 7–18% between the experimental and control reactors could lie within the limits of the statistical error. Thus, the Student’s *t*-test was used to analyze the effect of formate and folate. It confirmed that the addition of formate had no effect on the total dN (*t*-test, *p* = 0.1706), while the effect of the added folate was statistically significant (*t*-test, *p* = 0.0001).

#### Dynamics of the Composition of Microbial Community

**FIGURE 3:**
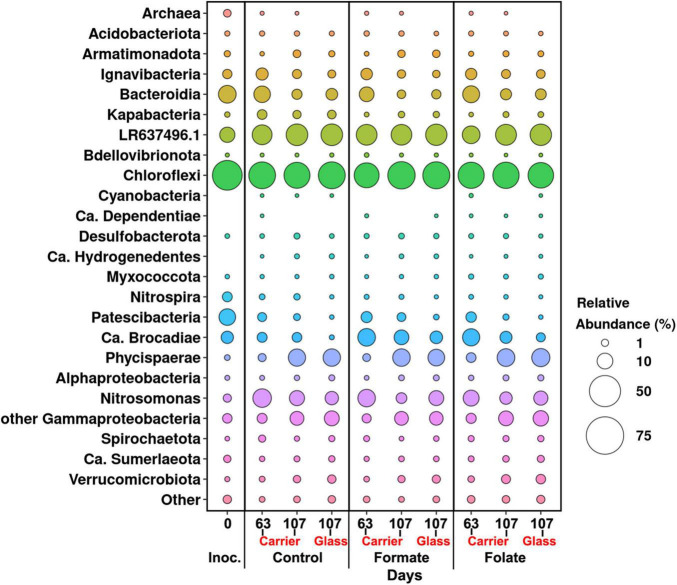
Overall microbial diversity in the inoculum and the biomass on the carrier and on the walls of the reactors (glass) before (the 63rd day) and after (the 107th day) the additives supplied and the ammonium load increased (the first run of the reactors) as revealed by high-throughput sequencing of the 16S rRNA gene fragments.

In general, the following representatives of four phyla dominated both in the inoculum and in the biofilms on the carrier and walls of the reactors regardless of additives: *Chloroflexi*, *Bacteroidetes*, *Planctomycetes*, and *Proteobacteria*. One of the most abundant OTU detected in the inoculum and in the biomass of all reactors (OTU 1) contributed 9–21% to the total number of 16S rRNA gene sequences. It was assigned to the *Bacteroidetes/Chlorobi* group but was only distantly related to cultured species and had maximal sequence identity (98.69%) with uncultured bacterium partial 16S rRNA gene (GenBank accession no. LR637496.1) obtained from a wastewater treatment system. A minor part of the community was formed by the representatives of the phyla *Acidobacteria*, *Armatimonadetes*, *Nitrospirae*, *Spirochaetes*, *Verrucomicrobia*, and *Patescibacteria* groups. The relative abundance of *Archaea* in the *inoculum* was 1.25%, showing the presence of hydrogenotrophic and acetoclastic methanogens of the genera *Methanothermobacter* and *Methanothrix*, respectively. It did not exceed 0.06% in biofilms on the carrier during the entire experiment and was not found on the glass (Figure 3). *Chloroflexi* was the dominant phylum (30–45%) and was represented mainly by the bacteria of the *Anaerolineae* class (filamentous anaerobic organotrophs), which are often detected in anammox reactors. Among the representatives of the phylum *Bacteroidetes*, the bulk of the sequences belonged to organotrophic members of the orders *Ignavibacteriales* and *Cytophagales*. Representatives of the class *Betaproteobacteria* belonged to the genera *Nitrosomonas* (stage I nitrifiers), *Comamonas*, and *Thauera*. Denitrifiers were found among the members of the latter two genera.

**FIGURE 4:**
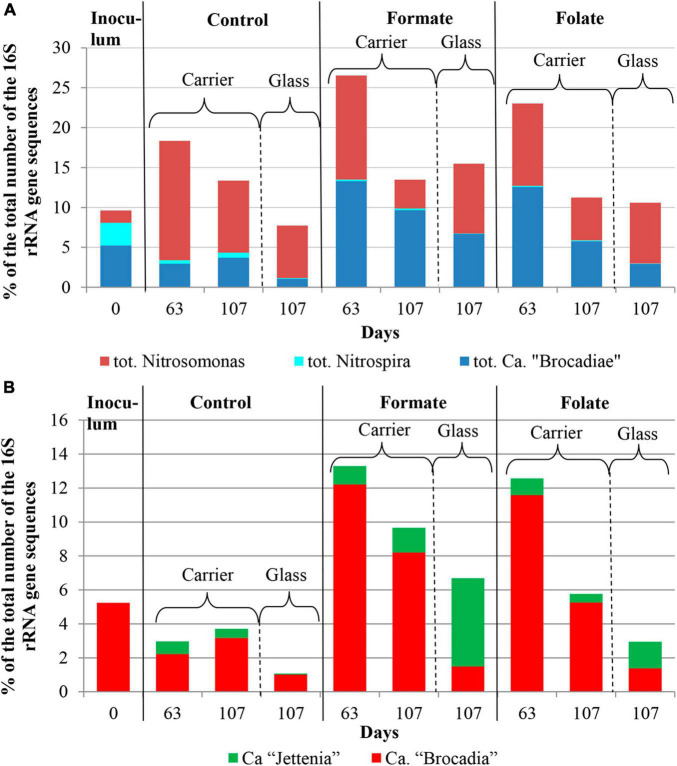
The overall abundance of stage I (genus *Nitrosomonas*) and stage II (genus *Nitrospira*) nitrifiers and anammox bacteria **(A)** and the relative abundance of members of the genera *Ca.* “Jettenia” and *Ca.* “Brocadia” **(B)** in the inoculum and the biomass on the carrier and on the walls of the reactors (glass) before (the 63rd day) and after (the 107th day) the additives supplied and the ammonium load increased (the first run of the reactors).

The relative abundance of sequences affiliated with members of the genus *Nitrosomonas* increased from 1.6% (in the inoculum) to 10–15% in the biofilms on the carrier in the period before the ammonium concentration was increased and additives were supplied. After the nitrogen loading was increased, their abundance on the carrier was decreased: the minimum abundance of 3.6% was found in the formate reactor and the maximum of 9% in the control one (Figure 4A). At the same time, it was found that *Nitrosomonas* sequences dominating in the inoculum (OTU 4; 1.24%) were displaced after 2 months of the operation of the reactors by other members of this genus (OTU 6; 11% in the reactors vs. 0.3% in the inoculum). The OTU 4 sequences again became dominant in the reactors after the regime was changed, and the OTU 6 fraction dropped to almost zero, which may be due to the influence of the incoming ammonium concentration.

In addition to the biomass immobilized on the carrier, another type of biofilms developed on the inner surface of the walls of the reactors (referred to as glass). In contrast to the carrier, on which a dense bright orange biomass was formed, biofilms on the glass were slightly pink in color, thin and transparent, easily separated from the glass, and floated to the surface (Figure 1B). These biofilms began to form after the load was increased and were the weakest in the control reactor. The differences between the relative abundance of microbial taxa were more pronounced in the biofilms on the glass than on the carrier (Figure 3). The relative abundance of *Nitrosomonas* sequences varied on the glass from 6.6 to 8.7% and was maximal in the formate reactor (Figure 4A).

The relative abundance of stage II nitrifiers of the genus *Nitrospira* reached 2.8% in the inoculum and decreased to <1% on the carrier and <0.1% on the glass. The maximum share of stage II nitrifiers (0.4–0.6%) was observed in the control reactor on the carrier (Figure 4A).

Representatives of the phylum *Planctomycetes* were among the most abundant members of the community (5.6% in the *inoculum* and 4.4–21.6% in the reactors). Of these, the relative abundance of sequences affiliated with uncultured representatives of the *Phycisphaerae* class was 0.4% in the inoculum, 1.3–2.6% in the reactors before the regime was changed, and 10.5–13.4% after the regime was changed. The rest was accounted for anammox bacteria of the *Ca*. “Brocadiae” family.

In the inoculum, the target family of *Ca*. “Brocadiae” was represented by sequences (5.2%) affiliated with anammox bacteria of the genus *Ca.* “Brocadia” (Figure 4B). They dominated in the biofilms on the carrier in all reactors, regardless of the load and the additives. The sequences related to anammox bacteria of the genus *Ca* “Jettenia” were also found. Their relative abundance was about 1% before the ammonium concentration was increased (Figure 4B). In the reactor, which became the control, the relative abundance of anammox bacteria after 2 months of operation decreased for unknown reasons almost two times compared to the inoculum (from 5.2 to 3%); the share of stage I nitrifiers, on the contrary, increased 10 times (from 1.6 to 15%). These data are consistent with the results of chemical measurements that recorded ejections of nitrite and nitrate in this reactor during the commissioning stage (Figures 2B,C). After the load increased, the abundance of anammox bacteria in the carrier biofilms of the control reactor slightly increased (3 vs. 3.7%) together with a decrease in the share of stage I nitrifiers (15 vs. 9%). In general, a decrease in the relative abundance of anammox bacteria and stage I nitrifiers was recorded in the carrier biofilms of both experimental reactors after an increase in the load and additive supply. The share of stage II nitrifiers remained at a low level. In the formate reactor, the abundance of the sequences related to *Ca.* “Brocadia” decreased 1.5 times and to *Ca.* “Jettenia” oppositely increased. In the folate reactor, the most significant reduction (2.2 times) in the abundance of *Ca.* “Brocadia” was observed, as well as a two times decrease in the share of *Ca.* “Jettenia” (Figure 4B). Thus, with the introduction of formate against the background of an increase in the ammonium concentration, the *Ca*. “Jettenia” contribution in carrier biofilms increased maximally (three times compared with the control and folate reactors) with a general decrease in the abundance of *Ca.* “Brocadia.”

In contrast to the carrier, representatives of the genus *Ca.* “Jettenia” dominated (5.2%) in biofilms on the glass of the formate reactor; the relative contribution of *Ca.* “Brocadia” was only 1.5%. The ratio between *Ca.* “Brocadia” and *Ca.* “Jettenia” on the glass of the folate reactor was 1:1, and in the control reactor, *Ca*. “Brocadia” dominated (Figure 4B). Thus, the stimulation of anammox bacteria of the genus *Ca.* “Jettenia” with formate was revealed, and spatial separation of two species of anammox bacteria inside one reactor was observed.

### The Second Run of the Reactors

#### Dynamics of the Concentration of Mineral Nitrogen Species

**FIGURE 5:**
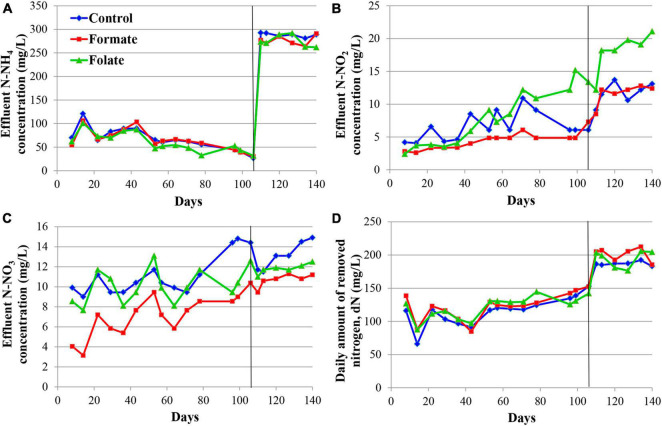
Dynamics of the concentrations of nitrogen compounds in the effluent during the second run of the reactors (milligrams per liter): concentrations of N–NH_4_ **(A)**, N–NO_2_ **(B)**, N–NO_3_ **(C)**, and daily amount of removed nitrogen (dN) **(D)**. A black vertical line separates periods before and after the ammonium load increased.

A mixed biomass sample from the first run was stored for 3 months at +4°C, followed by its activation for a week in the described mode without the supply of additives under an input ammonium concentration of 200 mg N–NH_4_/l. After the biomass activation, it was used as an inoculum for the second run of three parallel reactors. Formate was added to the first reactor and folate to the second one; both additives were supplied immediately upon a start-up. The third reactor was a control one (no additives). The ammonium concentration was increased in all reactors from 200 to 500 mg N–NH_4_/l after 3.5 months of operation. Figure 5 presents data on the dynamics of the concentration of nitrogen compounds in the effluent. The reactors were similar in terms of the concentrations of residual ammonium and dN (Figures 5A,D). The difference between the reactors was revealed when comparing the dynamics of nitrite and nitrate concentrations (Figures 5B,C). With the addition of formate, the average concentrations of nitrite (4.4 ± 1.3 and 11.6 ± 1.6 mg N–NO_2_/l before and after increasing the concentration of incoming ammonium, respectively) and nitrate (7.1 ± 2.1 and 10.7 ± 0.7 mg N–NO_3_/l) were expectedly lower than in the folate and control reactors, which could be associated with the DNRA process by anammox bacteria in the presence of formate. The concentration of nitrite steadily increased (from 2.4 to 21.1 mg NO_2_/l) in the folate reactor during the entire experiment. In the control reactor, the concentration of nitrate began to increase (from 10 to 15 mg N–NO_3_/l) by the end of the experiment. These variations, however, did not affect dN in both reactors. Before the load was increased, the dN was 115.3 ± 21.4 mg N/l (average value over the entire period) in the control reactor, 123.2 ± 20.0 mg N/l in the formate one, and 121.8 ± 16.6 mg N/l in the folate reactor. The formate reactor slightly pulled ahead in terms of dN after the concentration of incoming ammonium was increased: 201.4 ± 10.2 mg N/l (in the formate reactor) vs. 195.0 ± 12.9 mg N/l (in the folate reactor) and 186.8 ± 3.1 mg N/l (in the control) (Figure 5D). In general, the dN values in the first and second runs differed a little. However, in the second run, folate had no effect on dN (*t*-test, *p* = 0.0427 and *p* = 0.1943 before and after the load increased, respectively), while formate, on the contrary, had a stimulating effect (*t*-test, *p* = 0.0026 and *p* = 0.0082) compared to the control.

#### Dynamics of the Composition of Microbial Community

**FIGURE 6:**
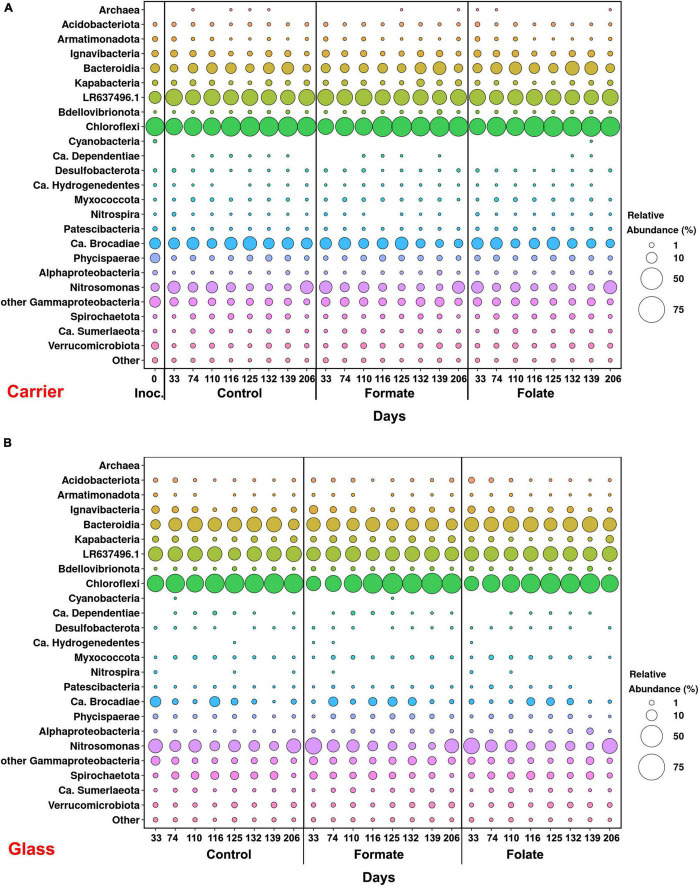
The overall microbial diversity in the inoculum and the biomass on the carrier **(A)** and on the walls of the reactors (glass) **(B)** before (0–74 days) and after (110–206 days) the additives supplied and the ammonium load increased (the second run of the reactors) as revealed by high-throughput sequencing of the 16S rRNA gene fragments.

**FIGURE 7:**
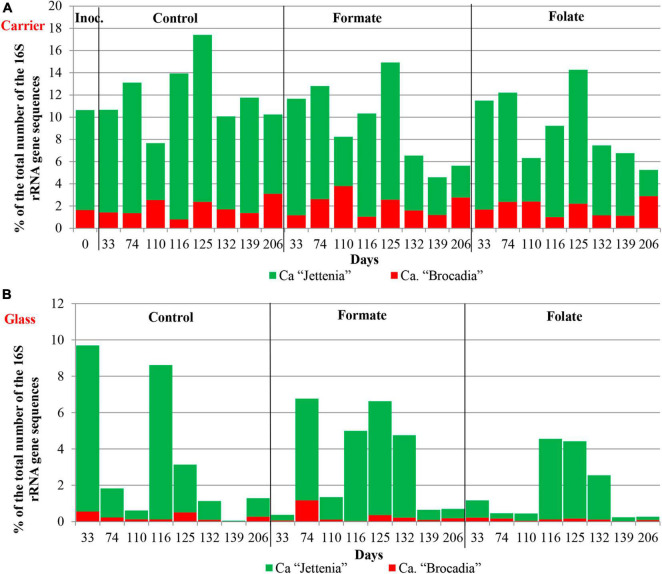
The relative abundance of members of the genera *Ca.* “Jettenia” and *Ca.* “Brocadia” in the inoculum and the biomass on the carrier **(A)** and on the walls of the reactors (glass) **(B)** before (0–74 days) and after (110–206 days) the additives supplied and the ammonium load increased (the second run of the reactors).

**FIGURE 8:**
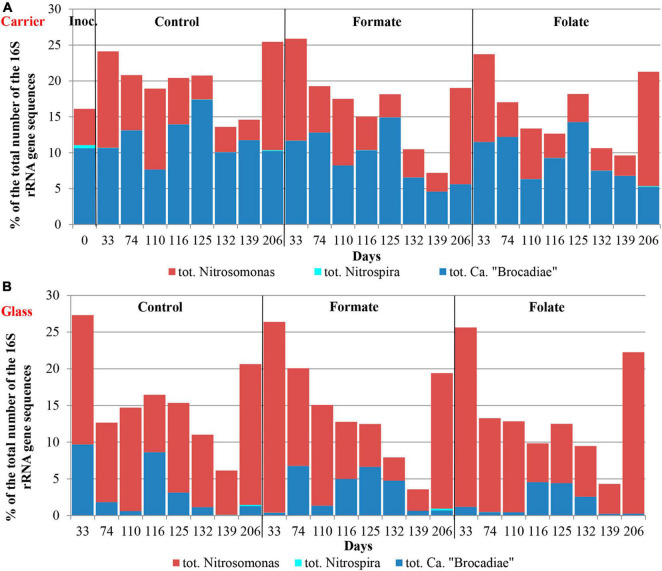
The overall abundance of stage I (genus *Nitrosomonas*) and stage II (genus *Nitrospira*) nitrifiers and anammox bacteria (*Ca.* “Brocadiae” family) in the inoculum and the biomass on the carrier **(A)** and on the walls of the reactors (glass) **(B)** before (0–74 days) and after (110–206 days) the additives supplied and the ammonium load increased (the second run of the reactors).

Figure 6 presents the dynamics of the composition of the microbial community in two types of biofilms (on the carrier and the glass). The overall composition of the community did not differ from that in the first run. There was also no difference in the composition of the community between different reactors (formate, folate, and control). However, the biofilms on the carrier and the glass differed slightly in terms of relative abundances of some taxa. No archaea were found in the biofilms on the glass. The abundance of representatives of the phyla *Nitrospirae*, *Ca.* “Hydrogenedentes,” and the *Ca.* “Brocadiaceae” family decreased, and the relative abundance of representatives of the phylum *Spirochaetes*, class *Bacteroidia*, and stage I nitrifiers of the genus *Nitrosomonas* increased in biofilms on the glass compared to the carrier (Figures 6A,B). The results on the ratio of anammox bacteria of the genera *Ca.* “Brocadia” and *Ca.* “Jettenia” in the community were of interest. At the first run, the anammox bacteria in the inoculum were represented almost exclusively by the genus *Ca.* “Brocadia.” During the experiment, representatives of the genus *Ca.* “Jettenia” came to light, and their relative abundance increased with the addition of formate (Figure 4B). A shift of *Ca.* “Brocadia” to *Ca.* “Jettenia” was documented in the inoculum after the biomass storage, and *Ca.* “Jettenia” spp. dominated in the reactors during the second run (Figure 7). In the inoculum for the second run, the relative abundance of stage I nitrifiers (*Nitrosomonas*) was two times lower than that of anammox bacteria (5.4 vs. 10.6%), stage II nitrifiers (*Nitrospira*) did not exceed 0.5% (Figure 8A), and the ratio of *Ca.* “Brocadia” to *Ca.* “Jettenia” was 1:5.6 (Figure 7A). During the operation of the reactors, anammox bacteria predominated among three target microbial groups on the carrier (Figure 8A), with the absolute dominance of sequences affiliated with representatives of the genus *Ca.* “Jettenia” (Figure 7A). The overall share of anammox bacteria on the carrier was higher in the control than in both experimental reactors (Figure 8A). The abundance of *Ca.* “Jettenia” decreased on the carrier by two times in all reactors immediately after the increase in nitrogen load; the share of *Ca.* “Brocadia” oppositely increased in the formate reactor (Figure 7A).

In contrast to the carrier, stage I nitrifiers dominated on the glass (Figure 8B). The abundance of anammox bacteria of the genus *Ca.* “Brocadia” did not generally exceed 1% in all reactors; the proportion of *Ca.* “Jettenia” ranged from 0.03 to 9% (Figure 7B). High temporal fluctuations in the relative abundance of anammox bacteria were observed. Their lowest share was recorded in the folate reactor (Figure 8B). Anammox bacteria of the genus *Ca.* “Brocadia” were almost completely washed out of the glass in all reactors by the end of the experiment; the smallest abundance of *Ca.* “Brocadia” spp. was observed in the folate reactor (Figure 7B).

Thus, the biomass storage conditions turned out to be unfavorable for the anammox bacteria of the genus *Ca.* “Brocadia” or, conversely, are favorable for the development of *Ca.* “Jettenia,” which resulted in an interspecies shift. Potential factors affecting the ratio between the two genera of anammox bacteria in the community could also be, besides the additives (formate and folate), DO, temperature, and nitrogen loading.

#### Comparative Study of *Ca*. “Brocadia” and *Ca*. “Jettenia” Genomes Derived From the Formate Reactor (The Second Run)

A complete metagenome, obtained from the biomass on the carrier of the formate reactor, was sequenced to assemble the composite genomes of the dominant members of the community. A total of 20.7 Gb was sequenced using Illumina technology and 3.1 Gb with Nanopore technology. The obtained sequences were assembled into contigs, which were binned into 39 MAGs with a CheckM assembly completeness of more than 80%. These MAGs accounted for 87% of the whole metagenome (Supplementary Table 1).

Phylogenetic identification of MAGs using genome-wide search in the GTDB database (Parks et al., 2018) revealed that four phyla dominated in the community: *Bacteroidetes* (36% of the whole metagenome), *Chloroflexi* (19%), *Proteobacteria* (13%), and *Planctomycetes* (11%). A minor part of the MAGs belonged to the phyla *Armatimonadetes* (2.6%), *Verrucomicrobia* (2.2%), *Ca.* “Omnitrophica” (1.3%), and *Spirochaetes* (0.1%). Among the anammox bacteria, two MAGs affiliated with the members of the genera *Ca.* “Jettenia” and *Ca.* “Brocadia” were obtained. The first MAG, designated Bin49, which accounted for 1.7% of the whole metagenome, belonged to the genus *Ca.* “Jettenia.” It was assembled into a single circular contig of 3,822,769 bp and had a 32.8 × average sequencing coverage. CheckM estimated its completeness in 95.6% with 1.65% possible contamination (redundancy). The second MAG, designated Bin9, which accounted for 3.3% of the whole metagenome, belonged to the genus *Ca.* “Brocadia.” It was also assembled into a single circular contig of 4,046,273 bp and had a 62.2 × average sequencing coverage. CheckM estimated its completeness in 100% with 2.75% of possible contamination (redundancy). Thus, both MAGs satisfy the proposed criteria for high-quality MAGs (completeness of >90% with contamination of <5% and the presence of all rRNA genes; Bowers et al., 2017). The 16S rRNA gene sequence of Bin49 was 99.28% identical to those of *Ca.* “Jettenia asiatica” (AB973443; Quan et al., 2008) and that of Bin9 was 100% identical to those of *Ca.* “Brocadia fulgida” (DQ459989; Kartal et al., 2004). These MAGs are the first complete ring genomes of anammox bacteria of these species.

To study the differences between two genomes of anammox bacteria and to identify the mechanisms of their competition, gene representation profiles were compared using the Clusters of Orthologous Groups of proteins (COG) database (Table 1). The analysis of the gene representation in each COG class showed that there are significantly more genes belonging to the categories of “cell motility,” “defense mechanisms,” and “mobilome: prophages and transposons” in the Bin 9 (*Ca.* “Brocadia”) genome than in the Bin 49 (*Ca.* “Jettenia”). Complete pathways for bacterial chemotaxis and flagellar assembly were found in the Bin 9 genome. These genes were absent in the Bin 49 genome; however, *Ca.* “Jettenia” sp. could form pili and use them to facilitate the attachment of cells to the walls of the reactor. Also, slightly more genes in the Bin 49 genome than in the Bin 9 fall into the categories “energy production and conversion;” “amino acid transport and metabolism;” “coenzyme transport and metabolism;” “posttranslational modification, protein turnover, and chaperones;” “signal transduction mechanisms;” “cell wall/membrane/envelope biogenesis;” and “extracellular structures.” A full set of genes for the synthesis of gas vesicles was discovered in the Bin 49 genome. All genes responsible for the anammox process were found in both genomes (Table 1).

**TABLE 1:** Comparative analysis of functional gene numbers on the COG class level between *Ca.* “Brocadia” and *Ca.* “Jettenia” genomes.

Category	Number of genes	
	*Ca.* “Jettenia” Bin.49	*Ca.* “Brocadia” Bin.9
RNA processing and modification	1	1
Energy production and conversion	157	134
Cell cycle control, cell division, and chromosome partitioning	40	39
Amino acid transport and metabolism	150	139
Nucleotide transport and metabolism	69	64
Carbohydrate transport and metabolism	95	91
Coenzyme transport and metabolism	169	155
Lipid transport and metabolism	62	68
Translation, ribosomal structure, and biogenesis	179	173
Transcription	64	61
Replication, recombination, and repair	102	101
Cell wall/membrane/envelope biogenesis	226	202
Cell motility	31	60
Posttranslational modification, protein turnover, and chaperones	134	108
Inorganic ion transport and metabolism	116	115
Secondary metabolite biosynthesis, transport, and catabolism	24	23
General function prediction only	192	176
Function unknown	54	54
Signal transduction mechanisms	160	145
Intracellular trafficking, secretion, and vesicular transport	41	33
Defense mechanisms	101	133
Extracellular structures	29	17
Mobilome: prophages and transposons	13	117
Cytoskeleton	3	5
Nitrogen transporters		
focA	4	5
NarK	1	1
AmtB	8	6
Anammox pathway		
nirK	2	1
Hao	10	6
HzsABC	4	3
Hdh	1	2
nrfA	1	1

### Co-occurrence and Mutual Exclusion Among Various Microorganisms in the Anammox Community

**FIGURE 9:**
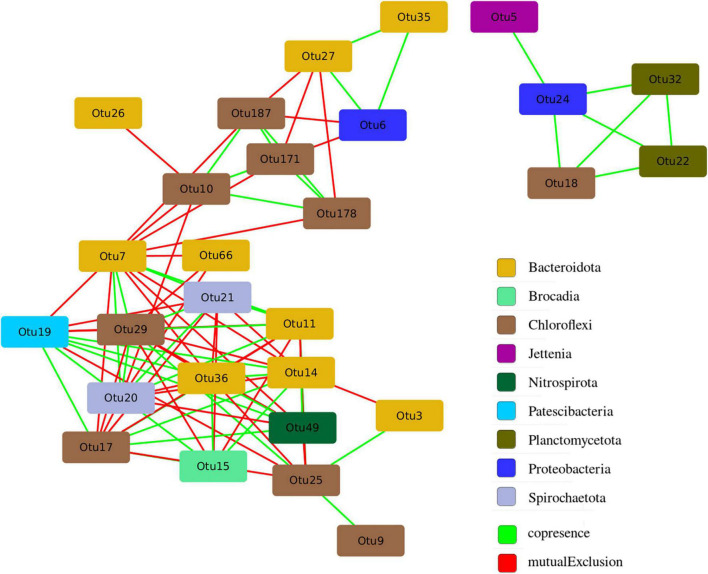
Co-occurrence and mutual exclusion among various microorganisms in the studied anammox community.

The structure of microbial community is not accidental and is closely related to the functional differentiation of microorganisms in the studied ecological niche. A network analysis was carried out in order to identify the patterns of interaction between groups of microorganisms in the anammox community. The analysis made it possible to identify co-occurrence and mutual exclusion among various microorganisms in the community (Figure 9).

All of 28 OTUs with a representation of more than 2% in at least one of the studied samples had statistically significant connections with other OTUs, the maximum number of which was 14 (Supplementary Table 2). It is assumed that OTUs with a large number of connections (“degree”) are key players in the entire community (Berry and Widder, 2014). The largest number of links was found for OTU 7, related to members of the order *Sphingobacteriales*. The proportion of sequences related to members of the order *Sphingobacteriales* was significantly higher in the samples taken from glass, which indicates their key role in the formation of biofilm on the walls of the bioreactor. Among the anammox bacteria represented by the genera *Ca.* “Jettenia” (OTU 5) and *Ca.* “Brocadia” (OTU 15), the latter OTU 15 had more connections and it is included in the main hub of organisms (Figure 9). At the same time, *Ca.* “Brocadia” had a positive relationship with heterotrophic bacteria of the phyla *Bacteroidetes* (OTU 36 and OTU 14) and *Chloroflexi* (OTU 17) widespread in wastewater treatment plants and with the bacteria of the *Patescibacteria* group. For the second anammox genus *Ca.* “Jettenia” (OTU 5), only one positive association with the denitrifying bacterium of the genus *Denitratisoma* was found.

## Discussion

Formate serves as an additional substrate for anammox bacteria (Kartal et al., 2007, 2008; Lawson et al., 2020) and folate being a secondary metabolite of different proteobacteria, can potentially increase the rate of nitrogen removal (Zhao et al., 2018). Thus, it was suggested that the addition of formate and folate would have a stimulatory effect on anammox bacteria, which in turn would lead to the stability of the anammox process under conditions of increased ammonium loads, i.e., it would help overcome a stress factor. A weak stimulatory effect of folate on dN was found during the first run (65–110 days). Formate did not affect the daily nitrogen removal, but its addition led to a decrease in the concentration of nitrate in the effluent compared to the control, which may indicate the progress in DNRA process by anammox bacteria (Kartal et al., 2007). Conversely, a weak stimulatory effect of formate and no effect of folate on dN were found during the second run (0–140 days). The miscellaneous influence of folate and formate on dN during the first and second runs was associated with a different effect of these compounds on the different genera of anammox bacteria that dominated in the community.

Among anammox bacteria, an absolute dominance of representatives of the genus *Ca.* “Brocadia” was revealed in the inoculum and the biomass on the carrier during the first run. Representatives of the genus *Ca.* “Brocadia” often gain an advantage over other anammox bacteria in various reactors, due to their significantly higher maximum specific growth rates (Zhang et al., 2017a,b, 2021). The highest μ_max_ among all known anammox bacteria was exhibited by the species *Ca.* “B. sinica” and *Ca.* “Brocadia sp.40” (Zhang and Okabe, 2020). The μ_max_ values were determined as 0.33 and 0.18 1/day for immobilized cells of anammox bacteria *Ca.* “B. sinica” and *Ca.* “J. caeni,” respectively (Zhang et al., 2017a). The additives (formate and folate) stimulated an increase in the relative abundance of sequences affiliated with representatives of the genus *Ca.* “Jettenia” and served as a trigger for the subsequent shift of the main anammox genus in the community. Shifts between different anammox bacteria have previously been reported, and the existence of genus- or species-specific niche differentiation and competition caused by differences in maximum specific growth rates, affinities to limiting substrates (ammonium and nitrite), susceptibility to various compounds, or VFA utilization has been suggested (Zhang et al., 2021). In the present study, the addition of formate clearly stimulated the growth of anammox bacteria of the genus *Ca.* “Jettenia.” However, the niche on the carrier has been already occupied by representatives of the genus *Ca.* “Brocadia,” and *Ca.* “Jettenia” failed in outcompeting them. It was suggested that an important role in further proliferation of anammox bacteria of the genus *Ca.* “Jettenia” in the reactor was played in terms of high adhesion capability and greater DO resistance compared with *Ca.* “Brocadia.” This allowed *Ca.* “Jettenia” to anchor and form biofilms on the inner surface of the walls of the reactor (“plastic glass” and polymethyl methacrylate). In the formate reactor, the share of representatives of the genus *Ca.* “Jettenia” in the biofilms on the glass reached 78% of anammox bacteria, while it did not exceed 15.5% in the biofilms on the carrier. Folate also stimulated the development of *Ca.* “Jettenia” on the glass, but to a lesser extent than formate. The relative abundance of the *Ca.* “Jettenia” on the glass was minimal in the control reactor. The results of the present study, however, contradicted the data of Ali et al. (2015) who showed that the anammox activity of *Ca.* “Jettenia” was inhibited by the addition of formate.

The ability of anammox bacteria of *Ca.* “Jettenia” to excessively produce extracellular polymeric substances (EPS) could play a key role both in their adhesion to glass and in protection from the toxic effects of DO. Aggregation and adhesion of anammox bacteria occur due to protein-rich EPS (Hou et al., 2015; Chen et al., 2019; Peeters and van Niftrik, 2019). Members of the genus *Ca.* “Jettenia” produce significantly more EPS proteins than *Ca.* “Brocadia,” *Ca.* “Kuenenia,” and *Ca.* “Scalindua” (Chen et al., 2019). Increased cell aggregation due to increased synthesis of EPS matrix in response to unfavorable environmental conditions was shown for representatives of the species *Ca.* “Jettenia caeni” (Guo et al., 2017). Anammox bacteria of *Ca.* “Jettenia,” releasing EPS in excess and enveloping cells with them, could thus protect themselves from the toxic effects of DO or other substances (Kallistova et al., 2020), which gives them an advantage over *Ca*. “Brocadia” and allows the spatial separation of these two genera in the same reactor. The success of *Ca.* “Jettenia” in the colonization of the walls of the reactors could also be due to their capability to synthesize type IV pili. It was shown that *Ca.* “Jettenia” served as initial colonizers, and type IV pili were responsible for the non-specific and irreversible adhesion to the surface (Xiao et al., 2021). Such ability to colonize smooth surfaces due to extra EPS production and formation of the pili could help *Ca.* “Jettenia” adapt to the competitive environment. In addition, biofilms on the carrier and the glass were influenced by different small-scale hydraulics, which, in turn, impacted the microbial structure and biodiversity (Niederdorfer et al., 2016). Obviously, the supply of ammonium and DO is higher in biofilms on the walls of the reactor than on the carrier; on the latter, substrate gradient and anaerobic zones exist [the stages of biofilm formation and stratification on the carrier are well described in Niederdorfer et al. (2021)]. At the same time, a better DO supply in biofilms on the walls of the reactor creates more favorable conditions for the development of stage I nitrifiers, and, accordingly, anammox bacteria on the glass are provided with nitrite better than in the biofilms on the carrier. This was confirmed by data on the relative abundances of stage I nitrifiers in biofilms on the glass and the carrier.

Anammox metabolism is supported by anoxic conditions or low oxygen concentrations (Strous et al., 1997; Egli et al., 2001; Dalsgaard et al., 2014; Cho et al., 2020). Quite a lot of studies, including transcriptome analyses, have been carried out in recent years to elucidate the effect of various DO concentrations on the activity of anammox bacteria. Most studies revealed a reversible inhibition of anammox bacteria by oxygen, and the inhibitory DO concentration varied over a wide range [reviewed by Seuntjens et al. (2018) and Cho et al. (2020)]. A complete inhibition of anammox activity both at DO < 0.12 mg/l (Strous et al., 1997; Egli et al., 2001; Oshiki et al., 2016; Seuntjens et al., 2018) and at much higher concentrations (1.0 mg/l) (Niederdorfer et al., 2021), as well as retention of activity at DO of 3–3.8 mg/l, was shown for communities enriched with *Ca.* “Brocadia” (Oshiki et al., 2011; Carvajal-Arroyo et al., 2013; Zekker et al., 2014). This variability could be caused by the inter-genera differences, adaptation to oxygen stress, protection by aerobic microorganisms, the existence of local hypoxic/anoxic zones, and testing procedures (Seuntjens et al., 2018 and the references therein). It has also been shown that anammox bacteria could retain some activity even under aerobic conditions, and the anammox process had a stronger tolerance to higher DO concentrations compared to the denitrification process (Wang L. et al., 2021). The mechanisms and genes involved in oxygen detoxification in anammox bacteria of genera *Ca.* “Brocadia” and *Ca.* “Kuenenia” were recently proposed (Ji et al., 2019; Yan et al., 2020; Wang H. et al., 2021). Data on the effect of DO on anammox bacteria of genus *Ca.* “Jettenia” are currently unavailable.

Once again, the inoculum was enriched by *Ca.* “Brocadia.” The addition of formate stimulated the growth of *Ca.* “Jettenia” in the reactor, which, due to their good adhesive capability and possibly to a higher DO resistance, anchored on the walls of the reactors being uncompetitive in the growth on the carrier in relation to *Ca.* “Brocadia,” so that *Ca.* “Brocadia” continued to prevail in biofilms on the carrier. However, after mixing the biomass from the three reactors and its storage at +4°C for 3 months, *Ca.* “Jettenia” became dominant (85% of anammox bacteria in the inoculum for the second run), i.e., a shift between two anammox bacteria occurred precisely at the stage of biomass storage. It can be hypothesized that the selective effect was exerted by the low storage temperature for a rather long time, to which *Ca.* “Jettenia” was more resistant compared with *Ca.* “Brocadia.” The literature data, however, do not support this hypothesis. According to previous studies, anammox bacteria belonging to these genera grow well in the temperature range of 20–45°C (Strous et al., 1999; Oshiki et al., 2011; Ali et al., 2015; Nikolaev et al., 2015; Kallistova et al., 2016), and it is representatives of the genus *Ca.* “Brocadia” that dominate in reactors at low temperatures, down to 5–6°C [reviewed in Tomaszewski et al. (2017) and Cho et al. (2020)]. They also maintain the activity in many freshwater environments at near 0°C, which may indicate their cold-acclimation capacity (Huo et al., 2020 and the references therein). There are little data on the effect of low temperature on anammox bacteria of the genus *Ca.* “Jettenia;” nevertheless, it has been shown that when the temperature drops from the optimum (35°C) to 25°C, *Ca.* “Brocadia” has an advantage over *Ca.* “Jettenia” due to the expression of more cold shock proteins and core enzymes (Huo et al., 2020).

Another selective factor that could lead to an interspecies shift in anammox bacteria is the concentration of ammonium and nitrite, the direct substrates of the anammox process. Ammonium is usually more abundant than nitrite in environments inhabited by anammox bacteria. Therefore, the availability of nitrite becomes more relevant to the niche differentiation among anammox bacteria (Zhang and Okabe, 2020). According to the Michaelis–Menten kinetics, a higher half-saturation constant (*K*_s_), a lower affinity to substrate, and a higher substrate concentration are needed to reach maximum growth rate. Thus, *K*_s_ is the key parameter for microbial growth and consequent niche differentiation. Anammox bacteria of the species *Ca.* “B. caroliniensis” and *Ca.* “B. fulgida” were shown to have the highest *K*_s_ values among anammox bacteria (including *Ca.* “Jettenia caeni”) for both NH_4_^+^ and NO_2_^–^ (Oshiki et al., 2016; Zhang and Okabe, 2020). This means that these *Ca.* “Brocadia” species have the lowest affinity to substrate among anammox bacteria and therefore prefer high substrate concentrations. It was found in experiments with three enrichment cultures of anammox bacteria (*Ca.* “Brocadia,” *Ca.* “Jettenia,” and *Ca.* “Kuenenia”) that *Ca.* “Jettenia” could proliferate only at low nitrogen loading rates (NLRs), i.e., low influent ammonium and nitrite concentrations, whereas *Ca.* “Brocadia” outcompeted the other two species at higher NLRs. Spatial distribution of *Ca.* “Jettenia” cells in the inner part of biofilms allowed authors to suggest that *Ca.* “Jettenia” prefers low-nitrite environments (Zhang et al., 2017b). An intrinsic growth kinetics difference between *Ca.* “Jettenia” and *Ca.* “Brocadia” can lead to the proliferation and domination of *Ca.* “Jettenia” under low NLRs in the presence of acetate (Zhang et al., 2017a,b, 2021). Indeed, a half-saturation constant (*K*_s_) for NH_4_ in three out of five known *Ca.* “Brocadia” species is higher than in *Ca.* “J. caeni” (Oshiki et al., 2016 and the references therein). The observed population shift from *Ca.* “Brocadia” to *Ca.* “Kuenenia” was suggested to be caused by the higher affinity for nitrite of representatives of *Ca.* “Kuenenia” (van der Star et al., 2008) as they have a much lower value of *K*_s_ for NO_2_ than *Ca.* “Brocadia” (Ding et al., 2013; Oshiki et al., 2016 and the references therein). However, in respect of *K*_s_ for NO_2_, the situation with *Ca.* “Brocadia” and *Ca.* “Jettenia” species is not so clear, as three out of five *Ca.* “Brocadia” species have *K*_s_ values for NO_2_ much lower or comparable to those of *Ca.* “J. caeni” (Oshiki et al., 2016 and the references therein). However, the value of *K*_s_ for NO_2_ for *Ca*. “Brocadia sinica” was shown to depend on the type of biomass. It is much lower for biomass in the form of planktonic cells than in the form of homogenized aggregates (Oshiki et al., 2013). It should be noted that there are anammox bacteria (e.g., *Ca.* “Kuenenia” and *Ca.* “Scalindua”) that have a higher affinity to the substrate than representatives of the genus *Ca.* “Jettenia” (Oshiki et al., 2016; Zhang and Okabe, 2020), and therefore, they could outcompete *Ca.* “Jettenia” under conditions of limited nitrogenous substrates (Xiao et al., 2021).

In the system we studied, the 16S rRNA gene sequence of *Ca.* “Brocadia” had 100% identity to those of *Ca.* “Brocadia fulgida” with the highest *K*_s_ value reported; thus, it should have an advantage at high substrate concentrations. On the contrary, *Ca.* “Jettenia” should potentially be more resistant than *Ca.* “Brocadia” to substrate starvation during long-term biomass storage. Then, there is a probability that most of *Ca.* “Brocadia” in the biomass died off during the storage with a deficiency of ammonium and nitrite in turn, and after being exposed to favorable conditions, *Ca.* “Jettenia” took advantage of this, enriched quickly, and further served as an inoculum for the second run. The results of the second run can confirm that *Ca.* “Brocadia” prefers higher ammonium concentrations than *Ca.* “Jettenia,” as a quick increase in the proportion of *Ca.* “Brocadia” and a decrease in the abundance of *Ca.* “Jettenia” were observed in all reactors after the ammonium load was increased from 177.8 to 444.4 mg N–NH_4_/l/day (Figure 7A). The opposite results, however, were demonstrated by Zhao et al. (2019), who proposed that *Ca.* “Brocadia” out-competed *Ca.* “Jettenia” under low nitrogen concentrations. The authors found that *Ca.* “Brocadia” genomes encoded the complete pathway for bacterial chemotaxis and flagellar assembly, a more complete two-component signal transduction system, and more redundant marker genes of nitrite reductase compared with *Ca.* “Jettenia” genomes. Altogether, this led to an advantage of *Ca.* “Brocadia” over *Ca.* “Jettenia” at low nitrogen concentrations, as they could improve the access to higher substrate concentrations by movement, they could sense and respond to nitrogen limitation by inducing changes in transcription, and they could adapt quickly and maintain the nitrogen metabolism stability under nitrite limitation. The higher competitiveness for nitrite of *Ca.* “Brocadia” led to its higher transcript activity and higher nitrogen removal rate (Zhao et al., 2019).

The selection of anammox bacteria species can also be influenced by other microorganisms of the community. Interspecies relationships based on spatial and substrate synergism and competition are established between microorganisms closely coexisting in anammox biofilms. Intraspecific interactions also take place according to the principle of quorum sensing and cross-feeding (Ding et al., 2013; Lawson et al., 2017; Zhao et al., 2018; Tang et al., 2019; Kuenen, 2020). For example, an exchange with secondary metabolites, contributing to EPS production and degradation, and thus to aggregation, and participation in nitrite loop were found between anammox bacteria and bacteria of the phyla *Armatimonadetes*, *Proteobacteria, Chloroflexi*, and *Chlorobi* (Zhao et al., 2018), as well as co-metabolism with *Patescibacteria* group (Hosokawa et al., 2021). It is assumed that one of the possible ecological roles of the bacteria of the *Patescibacteria* group in the anammox reactor is to provide lactate and formate to other coexisting bacteria, supporting their growth (Hosokawa et al., 2021). All these bacteria along with other typical members of the anammox communities were found in the present study. A comparison of the obtained sequences with those of culturable microorganisms with known functions in the community as well as with unculturable representatives for which metagenomics and metatranscriptomic data are available revealed bacteria that are directly involved in the nitrogen cycle, e.g., genera *Nitrosomonas* (stage I nitrifiers), *Nitrospira* (stage II nitrifiers), *Comamonas*, *Thauera*, and *Denitratisoma* (denitrifying bacteria). However, the vast majority of bacteria found were organotrophs. Their role in the anammox community most likely consists in the decomposition of dead cell biomass of activated sludge and EPS formed by anammox bacteria (Mardanov et al., 2016). Besides, according to metagenomics and metatranscriptomic studies available in the literature, most high-abundance heterotrophic members of the community detected (e.g., *Chloroflexi*, *Ignavibacteria*, and *Armatimonadetes*) have genes encoding different enzymes of nitrogen metabolism (Mardanov et al., 2019b). The network analysis also revealed the largest number of links for bacteria related to members of the order *Sphingobacteriales*. Members of this order are widespread in wastewater treatment bioreactors (Hu et al., 2012), especially in MBR-type bioreactors (Saunders et al., 2013; Zha et al., 2020). They produce glycosphingolipids, which provide a supportive and protective environment for biofilm dwellers (Al Ashhab et al., 2014). At the same time, the overall composition of the bacterial community remained relatively stable despite the supply of additives, the increase in the ammonium load, and the storage of biomass. Thus, it is not yet possible to link the effect of other microorganisms of the community to the prevalence of a particular anammox species.

The comparative genome analysis performed in this study allows one to speculate on the survival strategies of *Ca.* “Brocadia” and *Ca.* “Jettenia” spp., which can help explain the observed interspecies shift. The *Ca.* “Brocadia” genome encodes a more complete system of cellular chemotaxis and flagellar assembly than the *Ca.* “Jettenia” genome, which is consistent with the data of Zhao et al. (2019), who showed that all these genes were expressed at the RNA level. Mobility and chemotaxis functions allow members of the genus *Ca.* “Brocadia” to effectively navigate in a new environment and quickly occupy the most favorable niche. In addition, the large representation of genes for defense mechanisms, as well as phages and transposons, indicates that *Ca.* “Brocadia” encounters a large number of threats from other organisms of the community and can effectively compete with them. The strategy of *Ca.* “Brocadia” when environmental conditions change is to, if possible, “escape” from adverse effects, for example, to move into the inner layers of the microbial biofilm. If it is impossible to escape, then *Ca.* “Brocadia” are likely to die. The *Ca.* “Jettenia” immobility forces it to better adapt to environmental fluctuations, and on the spot, when it faces an adverse impact, it more or less successfully develops a suitable defense mechanism for the situation. This confirms the greater representation of the genes of categories “cell wall/membrane/envelope biogenesis” and “extracellular structures” in the *Ca.* “Jettenia” genome than in the *Ca.* “Brocadia” genome, which is consistent with the assumption that *Ca.* “Jettenia” can protect itself by the reinforcement of the outer wall, including the extra EPS formation, which not only improves its adhesion to various surfaces but also protects against increased concentrations of adverse substances (e.g., DO, nitrite, and ammonium). In addition, the *Ca.* “Jettenia” genome revealed a higher abundance of genes for energy transformation and catabolism (categories “energy production and conversion,” “amino acid transport and metabolism,” and “coenzyme transport and metabolism”) and for signal transduction and cell protection (categories “posttranslational modification, protein turnover, and chaperones” and “signal transduction mechanisms”) than the *Ca.* “Brocadia” genome. This may contribute to the survival of members of the genus *Ca.* “Jettenia” during the long-term low-temperature storage under starvation. Genes encoding the synthesis of gas vesicles were also discovered in the genome of *Ca*. “Jettenia.” Although gas vesicles are often regulated by light (Speth et al., 2017), it can be assumed that their formation can stabilize the position of *Ca.* “Jettenia” cells during biofilm formation on the walls of the reactors and can also protect them from overheating. So, the anammox component of the microbial community evidently exhibits high lability in dependence on the set of environmental conditions (Mardanov et al., 2016).

## Conclusion

From the results of the study, the following conclusions can be inferred:

• The addition of formate stimulates the growth of *Ca.* “Jettenia” spp. in the anammox community dominated by *Ca.* “Brocadia” spp. Abundant EPS production, which improves cell adhesion to various surfaces and protects the cell against increased concentrations of adverse compounds, could aid *Ca.* “Jettenia” spp. to gain a foothold on the wall of the reactor and build up biomass. Therefore, a spatial separation of competing anammox species is achieved within the same reactor.
• The higher resistance of *Ca.* “Jettenia” spp. to the starvation during biomass storage gives it an advantage over *Ca.* “Brocadia” spp. Altogether, this leads to an interspecies shift from *Ca.* “Brocadia” to *Ca.* “Jettenia” in the anammox community along with general sustainability of the anammox process, when the cultivation conditions (additives, load, and storage) change.
• Under the conditions of increased ammonium load, folate has a positive effect on dN in *Ca.* “Brocadia”-enriched community, and formate has a positive effect on dN in *Ca.* “Jettenia”-enriched community.
• Genome analysis suggests that *Ca.* “Brocadia” has the ability to quickly occupy the most favorable niche; it is competitive with other microorganisms of the community and escapes, if possible, from the adverse effects. The immobility of *Ca.* “Jettenia” forces it to better adapt to environmental fluctuations, and when faced with an adverse impact, it activates suitable defense mechanisms for adaptation and survival.

## Data Availability Statement

Raw 16S sequences can be found on the NCBI BioProject accession PRJNA556270.

## Author Contributions

AK, YN, and NP designed the study. VG, AD, JB, and AP performed the assembly, reactors maintenance, sampling, and chemical analyses. AB, EG, VK, NR, and AM performed the molecular analyses. AK analyzed the data and wrote the original draft of the manuscript. IZ performed the review and edited the manuscript. All authors have read and approved the submitted version.

## Conflict of Interest

The authors declare that the research was conducted in the absence of any commercial or financial relationships that could be construed as a potential conflict of interest.

## Publisher’s Note

All claims expressed in this article are solely those of the authors and do not necessarily represent those of their affiliated organizations, or those of the publisher, the editors and the reviewers. Any product that may be evaluated in this article, or claim that may be made by its manufacturer, is not guaranteed or endorsed by the publisher.
