# Supplementary material for: New Insight Into the Interspecies Shift of Anammox Bacteria Ca. “Brocadia” and Ca. “Jettenia” in Reactors Fed With Formate and Folate
Source: Front Microbiol. 2022 Feb 3;12:802201. doi: 10.3389/fmicb.2021.802201 (PMC8851195; doi:10.3389/fmicb.2021.802201)
Supplement: Supplementary file 1 [file Table_1.DOCX]

Supplementary Material

Table S1. General characteristics of MAGs.

| Genome/MAG | Phylogenetic assignment | Genome Completeness (ChekM) (%) | Total Length (bp) | Contigs | GC content (%) | Share in the whole metagenome |
| --- | --- | --- | --- | --- | --- | --- |
| bin.40 | *Armatimonadota; Chthonomonadales* | 96,3 | 4218862 | 84 | 64,5 | 0,88 |
| bin.18 | *Armatimonadota; Fimbriimonadaceae* | 94,09 | 2834224 | 32 | 61 | 0,52 |
| bin.77 | *Armatimonadota* | 95,45 | 6851397 | 171 | 70,6 | 1,17 |
| bin.61 | *Bacteroidota; Vicingaceae* | 99,36 | 2844059 | 94 | 36,1 | 0,20 |
| bin.47 | *Bacteroidota; Bacteroidia* | 93,17 | 2508525 | 41 | 31,6 | 0,22 |
| bin.57 | *Bacteroidota; Bacteroidia* | 87,98 | 2057426 | 51 | 33,6 | 0,15 |
| bin.54 | *Bacteroidota; Ignavibacteriaceae* | 96,72 | 4107554 | 68 | 44,1 | 0,84 |
| bin.13 | *Bacteroidota; Ignavibacteriaceae* | 99,16 | 3667572 | 217 | 34,7 | 0,58 |
| bin.68 | *Bacteroidota; Ignavibacteriaceae* | 93,01 | 3740111 | 78 | 47,7 | 0,54 |
| bin.5 | *Bacteroidota; Ignavibacteria* | 94,81 | 2450757 | 1 | 37,6 | 33,68 |
| bin.23 | *Bacteroidota; Kapabacteriaceae* | 96,99 | 2572411 | 37 | 49,7 | 0,30 |
| bin.20 | *Bdellovibrionota* | 91,01 | 3277816 | 23 | 54,9 | 0,49 |
| bin.42 | *Chloroflexota; Anaerolineales* | 91,82 | 3153642 | 135 | 40,3 | 0,20 |
| bin.12 | *Chloroflexota; Anaerolineales* | 93,64 | 4160486 | 8 | 56,5 | 16,58 |
| bin.35 | *Chloroflexota; Anaerolineales* | 86,36 | 2556596 | 326 | 52,8 | 0,12 |
| bin.14 | *Chloroflexota; Anaerolineales* | 89,09 | 2768657 | 282 | 61,5 | 0,18 |
| bin.6 | *Chloroflexota; Anaerolineae* | 94,5 | 5129891 | 39 | 60,7 | 1,62 |
| bin.8 | *Chloroflexota; Anaerolineae* | 84,5 | 4104062 | 839 | 56,4 | 0,18 |
| bin.2 | *Desulfobacterota_B; Binatia* | 86,56 | 3710102 | 328 | 69,6 | 0,24 |
| bin.63 | FEN-1099 | 97,74 | 5455653 | 28 | 63,7 | 0,71 |
| bin.21 | OLB16 | 98,28 | 4238192 | 101 | 54,6 | 0,99 |
| bin.50 | OLB16 | 97,85 | 6659496 | 446 | 51,7 | 0,36 |
| bin.41 | *Planctomycetota;* | 95,39 | 6563782 | 113 | 62,2 | 0,58 |
| bin.9 | *Planctomycetota;Ca. Brocadia fulgida* | 100 | 4046273 | 1 | 45,2 | 3,35 |
| bin.49 | *Planctomycetota; Ca. Jettenia* | 95,6 | 3822769 | 1 | 39,5 | 1,67 |
| bin.36 | *Planctomycetota; Phycisphaerales* | 97,66 | 3213163 | 1 | 68,9 | 4,50 |
| bin.60 | *Planctomycetota; Phycisphaerae* | 97,73 | 4211490 | 70 | 65,6 | 1,14 |
| bin.26 | *Planctomycetota* | 90,7 | 4092323 | 309 | 63,6 | 0,22 |
| bin.74 | *Proteobacteria; Rubrivivax* | 97,2 | 4508884 | 183 | 72,4 | 1,16 |
| bin.33 | *Proteobacteria; Nitrosomonas* | 98,56 | 3051633 | 2 | 48,8 | 6,91 |
| bin.64 | *Proteobacteria; Nitrosomonas* | 90,67 | 2166036 | 98 | 50,5 | 0,28 |
| bin.7 | *Proteobacteria; Nitrosomonas europaea* | 90,99 | 2120018 | 275 | 50,5 | 0,78 |
| bin.62 | *Proteobacteria; Rhodocyclaceae* | 91,78 | 3356234 | 6 | 66,5 | 3,51 |
| bin.69 | *Spirochaetota; Leptospiraceae* | 88,51 | 2818667 | 675 | 34,4 | 0,12 |
| bin.58 | Verrucomicrobiota; Opitutaceae | 96,62 | 4291206 | 35 | 66,7 | 0,76 |
| bin.39 | Verrucomicrobiota; Opitutaceae | 94,59 | 3689799 | 90 | 68,8 | 0,45 |
| bin.66 | Verrucomicrobiota; Didemnitutus | 99,32 | 4125092 | 39 | 67,2 | 0,68 |
| bin.30 | Verrucomicrobiota; Opitutaceae | 88,51 | 4908326 | 934 | 59,3 | 0,19 |
| bin.73 | Verrucomicrobiota; Prosthecobacter | 84,01 | 3670281 | 734 | 59,1 | 0,13 |
